# High multiple carriage and emergence of *Streptococcus pneumoniae* vaccine serotype variants in Malawian children

**DOI:** 10.1186/s12879-015-0980-2

**Published:** 2015-06-20

**Authors:** Arox W. Kamng’ona, Jason Hinds, Naor Bar-Zeev, Katherine A. Gould, Chrispin Chaguza, Chisomo Msefula, Jennifer E. Cornick, Benard W. Kulohoma, Katherine Gray, Stephen D. Bentley, Neil French, Robert S. Heyderman, Dean B. Everett

**Affiliations:** Microbes, Immunity and Vaccines, Malawi Liverpool Wellcome Trust Clinical Research Programme, Blantyre, Malawi; Biochemistry Department, University of Malawi, College of Medicine, Blantyre, Malawi; Institute of Infection and Global Health, University of Liverpool, Liverpool, UK; Division of Clinical Sciences, St George’s, University of London, London, UK; Microbiology Department, University of Malawi, College of Medicine, Blantyre, Malawi; International Centre for Insect Physiology and Ecology, Nairobi, Kenya; Pathogen Genomics, Wellcome Trust Sanger Institute, Cambridge, UK; Department of Medicine, University of Cambridge, Cambridge, UK; Malawi Epidemiology and Intervention Research Unit (MEIRU), Karonga, Malawi; Liverpool School of Tropical Medicine, Liverpool, UK

**Keywords:** *Streptococcus pneumoniae*, Serotype, Capsule biosynthesis, Multiple carriage

## Abstract

**Background:**

Carriage of either single or multiple pneumococcal serotypes (multiple carriage) is a prerequisite for developing invasive pneumococcal disease. However, despite the reported high rates of pneumococcal carriage in Malawi, no data on carriage of multiple serotypes has been reported previously. Our study provides the first description of the prevalence of multiple pneumococcal carriage in Malawi.

**Methods:**

The study was conducted in Blantyre and Karonga districts in Malawi, from 2008 to 2012. We recruited 116 children aged 0–13 years. These children were either HIV-infected (*N* = 44) or uninfected (*N* = 72). Nasopharyngeal samples were collected using sterile swabs. Pneumococcal serotypes in the samples were identified by microarray. Strains that could not be typed by microarray were sequenced to characterise possible genetic alterations within the capsular polysaccharide (CPS) locus.

**Results:**

The microarray identified 179 pneumococcal strains (from 116 subjects), encompassing 43 distinct serotypes and non-typeable (NT) strains. Forty per cent (46/116) of children carried multiple serotypes. Carriage of vaccine type (VT) strains was higher (*p =* 0.028) in younger (0–2 years) children (71 %, 40/56) compared to older (3–13 years) children (50 %, 30/60). Genetic variations within the CPS locus of known serotypes were observed in 19 % (34/179) of the strains identified. The variants included 13-valent pneumococcal conjugate vaccine (PCV13) serotypes 6B and 19A, and the polysaccharide vaccine serotype 20. Serotype 6B variants were the most frequently isolated (47 %, 16/34). Unlike the wild type, the CPS locus of the 6B variants contained an insertion of the *licD*-family phosphotransferase gene. The CPS locus of 19A- and 20-variants contained an inversion in the sugar-biosynthesis (*rmlD*) gene and a 717 bp deletion within the transferase (*whaF*) gene, respectively.

**Conclusions:**

The high multiple carriage in Malawian children provides opportunities for genetic exchange through horizontal gene transfer. This may potentially lead to CPS locus variants and vaccine escape. Variants reported here occurred naturally, however, PCV13 introduction could exacerbate the CPS genetic variations. Further studies are therefore recommended to assess the invasive potential of these variants and establish whether PCV13 would offer cross-protection. We have shown that younger children (0–2 years) are a reservoir of VT serotypes, which makes them an ideal target for vaccination.

## Background

Every year, approximately 800 000 deaths that occur in under-five children globally are attributed to invasive pneumococcal disease (IPD) [1]. The majority of these deaths are reported in Africa, with sub-Saharan Africa (SSA) bearing the greatest burden [1]. Carriage of *S. pneumoniae* is a pre-requisite of IPD, and for carriage to occur, the pneumococcus has to establish itself on the mucosal surfaces of the human nasopharynx [2].

Carriage plays a key role in pneumococcal transmission within the community [3]. An individual can carry one or more pneumococcal serotypes at any given time [4]. Carriage of two or more serotypes is defined as multiple carriage. With the increased availability of highly sensitive serotyping techniques such as the microarray, the full extent of global prevalence of multiple carriage is emerging [4]. Microarray determines pneumococcal serotype by detecting genes contained within the capsular polysaccharide (CPS) locus, which encode the polysaccharide capsule [5]. Microarray serotyping can be used to detect (i) multiple carriage; (ii) carriage of other bacterial species; (iii) the absence or presence of particular genes, *e.g.* antibiotic resistance genes; (iv) non-typeable serotypes (NTs); as well as (v) novel serotypes by detecting genetic variations at the CPS locus itself [6].

Multiple carriage is reported to promote genetic recombination, characterised by the acquisition of genetic elements from other microbes through transformation, transduction or conjugative transfer [7]. Given the pneumococcus is highly transformable and undergoes genetic recombination through horizontal gene transfer [8], recombination at the CPS locus may result in a change in serotype (capsule switching) [9] which could lead to vaccine escape [10]. Therefore, predicting the emergence of vaccine escape in a high IPD burden population such as in Malawi is crucial to understanding the likely long-term public health effect of pneumococcal vaccination.

In November 2011, Malawi introduced PCV13 in the national infant immunisation programme. Conjugate vaccines clearly reduce the burden of vaccine serotype disease and carriage [11]. However, the increase of non-vaccine serotypes (NVT) in carriage post-vaccination, has led to serotype replacement, which is a major concern [12]. In settings with a high diversity of pneumococcal carriage, serotype replacement may be exacerbated. Yet despite its importance in pneumococcal evolution, evidence of multiple carriage in Africa has not been well documented.

In the study reported in this article, we investigated the prevalence of multiple carriage and the degree of naturally occurring genetic variation at the CPS loci for important vaccine serotypes in Malawian children from 2008 to 2012, prior to country wide pneumococcal vaccine usage.

## Methods

### Study setting

The study was conducted at the Malawi-Liverpool-Wellcome Trust (MLW) Clinical Research Programme (Blantyre, Malawi). Children were recruited from households in the northern (Karonga) region, and from outpatients presenting at the Queen Elizabeth Central Hospital (QECH) in the southern (Blantyre) region of Malawi. QECH is a government-funded teaching hospital with 1 250 beds and provides clinical care to the population of about 1 million people [13]. Laboratory analyses were performed at MLW; St George’s Medical School, University of London (London, UK) and the Wellcome Trust Sanger Institute (WTSI, Cambridge, UK).

### Study participants

The study population comprised children ranging from 0 to 13 years. These children were recruited between 2008 and 2012 as part of a routine surveillance study investigating pneumococcal carriage and transmission in Malawi (Table 1). Consent to include the children in the study was obtained from parents or guardians. The samples collected were stored at −80 °C in the MLW archive, which has over 5000 nasopharyngeal samples. The samples analysed in this study were only from children who were carriers of *S. pneumoniae*, following initial screening based on colony morphology and optochin (Oxoid, UK) sensitivity. We randomly screened 189 samples, out of which 116 were positive for *S. pneumoniae*, representing a 61 % pneumococcal carriage rate. These children included both males (*N* = 68) and females (*N* = 48) who were either HIV negative (*N* = 72) or HIV positive (*N* = 44). HIV positive children were identified from the paediatric staging and ART outpatient clinics while the HIV negative controls were recruited from those children undergoing elective surgery for non-immune related conditions. Trained nurses were responsible for recruiting children into the study and collection of nasopharyngeal samples. The median age was significantly higher for HIV positive children (4.9 years) than for HIV negative children (1.4 years) (*p* = 0.001). However, there was no significant age difference between male and female children (*p* = 0.5).

**Table 1:** Characteristics of children enrolled in the study, Malawi, 2008-2012

		HIV status		
		Positive	Negative	Total
Gender	Male	68 % (*N* = 46)	32 % (*N* = 22)	68
	Female	54 % (*N* = 26)	46 % (*N* = 22)	48
IQR(Age)		1.4 (0.8-3.1)	4.9 (2.7-8.1)	

A summary of subjects by HIV status, gender and age (in years). The children were aged between 0 and 13 years. The interquartile range (IQR) for the median age is included

### Sample collection

Nasopharyngeal samples were collected from children using sterile Dacron-tipped nasopharyngeal swabs (Medical Wire and Equipment, Corsham, UK). The swabs were immediately preserved in skim milk, tryptone, glucose and glycerine (STGG) medium and then stored at −80 °C until further analysis.

### Culturing of *S. pneumoniae*

The frozen samples in STGG were briefly thawed to 4 °C. An aliquot (~10 μl) of the STGG sample was then cultured on sheep blood agar and gentamycin (SBG) selective media (MLW, Malawi). A presumptive identification of alpha-haemolytic streptococci as *S. pneumoniae* was made by colony morphology and optochin (Oxoid, UK) sensitivity by disc diffusion. Samples containing *S. pneumoniae* were then selected for further characterisation by a more sensitive molecular serotyping by microarray.

### DNA extraction

DNA extraction for microarray serotyping was performed as described previously [14]. Briefly, for each sample, the STGG containing tubes were briefly vortexed. Aliquots of the STGG solution from the swabs were diluted 1:10 and 1:100 using Brain Heart Infusion (BHI) Broth (Oxoid, UK). Each STGG dilution (50 μl) was plated onto a colistin-oxolinic acid-blood agar [15] plate (Oxoid, UK) and incubated overnight at 37 °C in 5 % CO_2_. DNA was extracted from whole-plate sweeps using QIAamp DNA Mini Kit (Qiagen, Germany). The DNA required for further analysis by whole-genome sequencing was extracted from single and morphologically distinct pneumococcal colonies on a culture plate, following the DNA extraction protocol above.

### Serotyping of *S. pneumoniae* by microarray

Molecular serotyping was performed using the protocol developed by the bacterial microarray group at St George’s (BμG@S) medical school: The BμG@S SP-CPS v1.4.0 microarray (bugs.sgul.ac.uk). Standard Agilent protocols for DNA labelling and hybridisation were used (Agilent, UK). The processed microarray slides were scanned using a high-resolution microarray scanner (Agilent, UK). Raw intensity data were extracted using Agilent Feature Extraction software (Agilent, UK). The output was analysed using an empirical Bayesian model to determine the pneumococcal serotypes present and their relative abundance [6]. In this study, pneumococcal serotypes were classified as either: PCV13 vaccine types (VT), non-vaccine types (NVT) or non-typeable (NT) strains [16].

### Sequencing whole genomes of *S. pneumoniae*

Whole-genome sequencing was performed on the Illumina HiSeq platform (CA, USA). The sequences were iteratively assembled using Velvet algorithm [17]. To determine genetic variations at the CPS loci, the reads were mapped against reference sequences for each serotype in order to obtain fine mapping using Burrows-Wheeler Aligner [18, 19]. The references used were for serotypes 6B [GenBank: CR931639], 19A [GenBank: CR931675] and 20 [GenBank: CR931679]. Single nucleotide polymorphisms (SNPS) were called using SAMtools, VCFtools and BCFtools [20]. In order to generate a multiple sequence alignment, we reduced the mapped binary alignment map (BAM) files to a consensus sequence for the CPS locus of each isolate used in the analysis. The sequence reads for the wild type and CPS locus variant pneumococcal isolates used for this analysis were deposited in the European Nucleotide Archive (ENA) of the European Bioinformatics Institute (EBI), accession numbers: [ERS003549, ERS006757, ERS006775, ERS012090, ERS012138, ERS012143, ERS050261, ERS050288, ERS050336, ERS050451, ERS096165, ERS096166, ERS096169, ERS096172, ERS096173].

### Phylogenetic analysis

A phylogenetic tree of the CPS locus variants was generated using only the polymorphic sites identified from the generated multiple sequence alignment, using RAxML version 7.8.6 with a Gamma model accounting for the rate heterogeneity among the sites [21]. To assess the reliability of the phylogeny, we performed 100 bootstrap replicates. Comparative analysis of the CPS sequences was performed using Artemis [22]. Previously characterised Malawian invasive serotype 6B isolates [10] were included in the phylogeny for comparison.

### Genetic recombination

Genetic recombination events were detected using the Geneologies Unbiased By Recombination in Nucleotide Substitutions (GUBBINS) tool [23]. Gubbins statistically determines genomic loci with atypically high number of variable sites, which is usually imported from exogenous sources. All the variable sites that were present in the inferred recombination regions were iteratively excluded from the sequence alignment. This generated a robust phylogeny whose evolution signals were not confounded by recombination.

### Statistical analysis

Microarray serotyping data were analysed using Stata 11 (StataCorp, College Station, TX, USA). Graphs were generated using R statistical software (www.R-project.org) and GraphPad Prism version 6.00 for Mac OS X (San Diego California USA, www.graphpad.com).

### Ethics

Ethical clearance to conduct this study was granted by the College of Medicine Research and Ethics Committee (COMREC), University of Malawi.

## Results

### Malawian children carry a broad range of pneumococcal serotypes

**Fig. 1:**
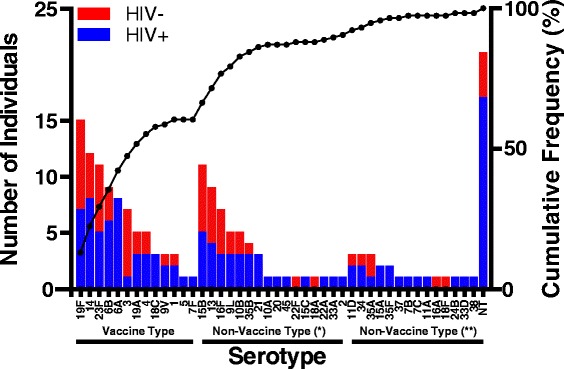
Serotype-specific pneumococcal carriage in Malawian children, determined by microarray. A total of 179 pneumococcal strains were detected from 116 nasopharyngeal swabs, comprising 43 distinct pneumococcal serotypes and non-typeable strains (NT). The blue and red bar graphs represent serotypes detected in HIV positive and HIV negative children respectively. The serotypes were classified as Vaccine Type and Non-Vaccine Type. The non-vaccine serotypes were subdivided into high (Non-Vaccine Type (*)) and low (Non-Vaccine Type (**)) invasive potential based on the global frequency of isolation from invasive disease [24]. Individuals carrying multiple serotypes are represented more than once. The line graph represents cumulative frequency of serotypes isolated and was used to estimate the proportion of Vaccine Type (60 %) and Non-Vaccine Type (40 %)

**Fig. 2:**
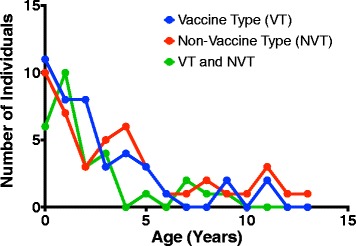
Age distribution for carriage of *S. pneumoniae* PCV13 (VT) and non-PCV13 (NVT) serotypes. A total of 116 pneumococcal isolates were analysed and categorised into three categories, namely carriage of only PCV13 (VT) serotypes (*N* = 42), carriage of only non-PCV13 (NVT) serotypes (*N* = 46) and carriage of both PCV13 and non-PCV13 (VT and NVT) serotypes (*N* = 28). The data for the VT or NVT carriers involved both single and multiple serotype carriage while the VT and NVT subjects were all carriers of multiple serotypes

The microarray identified 43 distinct serotypes in addition to NTs amongst the 179-pneumococcal strains isolated from the 116 children in this study (Fig. 1). VT serotypes represented 60 % of all the serotypes detected. The NVT serotypes accounted for 39 % of all the serotypes detected and these were classified into either high-invasive (30 %) or low-invasive (9 %) potential based on the reported global frequency of isolation from invasive disease [24]. The NT strains accounted for the remaining 1 % of the strains. We further investigated the impact of age on carriage of VT or NVT serotypes. Statistically, carriage of VT serotypes was significantly higher in the younger (0–2 years) than older children (71 %, 40/56 vs 50 %, 30/60; *p =* 0.028). However carriage of NVT serotypes was significantly higher in the older compared to younger children (50 %, 30/60 vs 29 %, 16/56; *p* = 0.028), suggesting that age had an impact on carriage of VT and NVT serotypes (Fig. 2). Serotype 1, which is the most isolated pneumococcal strain from invasive disease in Malawi [25], was only detected in 3 out of 116 children sampled, with two carriers in the 0–2 years age group and one carrier in older children.

### High prevalence of multiple carriage in Malawian children

**Fig. 3:**
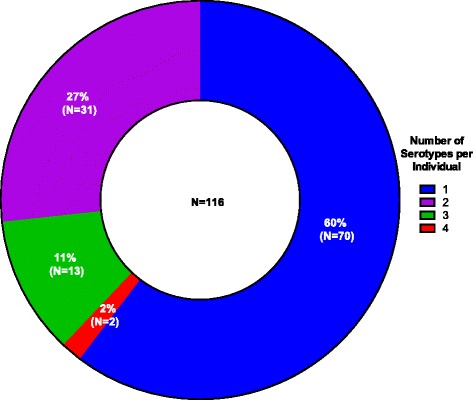
Multiple carriage of *S. pneumoniae* serotypes in Malawian children. Microarray was used to determine carriage of multiple pneumococcal serotypes in the nasopharynx of Malawian children. The overall frequency of multiple serotype carriage was 40 % (46/116), with co-colonising samples expressing two (27 %, 31/116)), three (11 %, 13/116) or four (2 %, 2/116) capsular types

We identified multiple carriage amongst 40 % (46/116) of the children’s samples analysed, with 27 % (31/116) of samples tested containing two serotypes, 11 % (13/116) containing three serotypes, and 2 % (2/116) containing four serotypes (Fig. 3). In those multiple carriage events, 61 % (28/46) co-carried at least one VT and an NVT, 15 % (7/46) carried VT only and 24 % (11/46) carried NVT only. The NVT pneumococcal strains were most abundant in 53.6 % (15/28) of multiple carriage events that occurred with VT strains. Since conventional serotyping methods are biased towards detection of the most abundant serotype, the VTs present at low abundance in multiple carriage events may not have been detected in these samples, which could have resulted in poor estimation of the vaccine coverage. The effect of HIV infection on multiple carriage was also investigated. Statistically, there was no significant difference in the prevalence of multiple carriage between HIV negative and HIV positive children (42 % vs 34 %, *p* = 0.34). The association between age and multiple carriage was also investigated. The children were divided into two groups: younger children aged 0–2 years (*N* = 56) and older children aged 3–13 years (*N* = 60). There was no statistically significant difference in multiple carriage between younger and older children (48 %, 27/56 vs 32 %, 19/60, *p =* 0.08). Overall, there was also no statistically significant variation in the median age for single or multiple carriers of pneumococcal serotypes (2.6 (1.5-5.0) vs 1.96 (1.1-3.9), *p =* 0.28), suggesting that age did not have an impact on multiple carriage.

### Evolution of the CPS genes belonging to pneumococcal vaccine serotypes

Highly divergent genes within the CPS loci associated with particular serotypes (6B, 19A and 20) were detected. Serotypes 6B and 19A are included in PCV13 and serotype 20 is included in the pneumococcal polysaccharide vaccine PPV23. These 3 variants accounted for over half of all the variants detected (34 % (16/37), 11 % (4/37) and 8 % (3/37) respectively). Given their abundance, these variants were sequenced and characterised to determine the genetic changes that had occurred at the CPS locus.

### How do the 6B CPS locus variants differ?

**Fig. 4:**
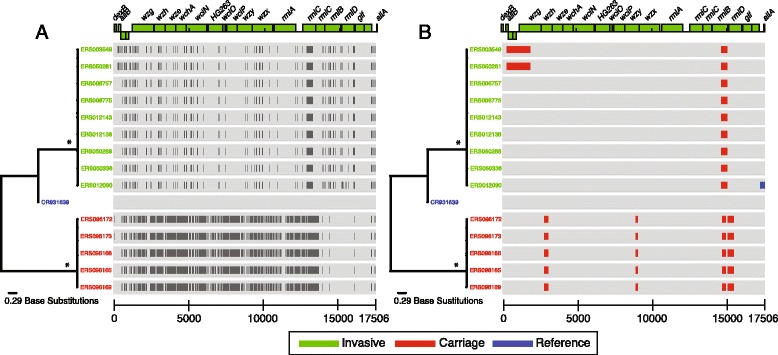
Genetic composition of the CPS sequences in wild type and variant 6B serotypes. The CPS locus sequence of serotype 6B [CR931639] was used as reference. The branches were coloured based on the type of the strain, either carriage (red), invasive (green), or reference (blue). **a** shows the distribution of SNPs within the CPS locus among the variants and invasive 6B serotypes. The black marks to the right of the terminal taxa represent location of SNPs within the CPS sequences. **b** shows the location of genetic recombination events. Unique recombination events are coloured in blue and shared (non-unique) events are coloured in red. Branches matched with an asterisk (*) represent nodes with a bootstrap value of 100 %

A phylogenetic analysis of Malawian 6B carriage variants and Malawian 6B invasive serotypes showed two genetically distinct clusters (Fig. 4a & b). Genetic diversity was determined through a single nucleotide polymorphisms (SNPs) analysis between the variant and wild type. Results showed that the CPS locus SNPs density was 50.7 SNPs/Kbp/strain for 6B variants, higher than the 9.0 SNPs/Kbp/strain in invasive 6B strains (Fig. 4a), showing a higher mutation rate in variants compared to the wild type strains.

### Variants demonstrate high genetic recombination

A recombination analysis showed four regions of recombination in all the 6B variants. These occurred in *wzg, wciO, rmlA* and *rmlC* genes (Fig. 4b), which encode for an integral membrane regulatory protein, putative ribitol transferase, glucose-1-phosphate thymidylyltransferase, and dTDP-4-keto-6-deoxy-D-glucose 3,5-epimerase respectively. All the invasive 6B serotypes showed recombination in the *rmlA* gene. All the recombination events in the 6B variants were ancestral meaning they occurred a long time ago and have since become fixed in the current circulating population. This fixation may be due to a selective advantage it confers to the variant related to survival or replication.

### A deletion within the *licD*-family phosphotransferase gene

**Fig. 5:**
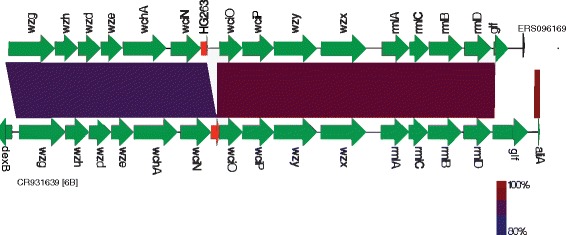
A comparison of the CPS locus sequence between serotype 6B variant and wild type. These variants were initially detected by microarray as having atypical CPS genes and were further analysed by whole-genome sequencing. A comparative analysis of the CPS locus sequences between variants of serotype 6B and reference sequence CR931639 was performed using Artemis. The divergent gene(s) for the variant are highlighted in red. The 6B variant [ERS096169] harboured an intact allele of the *licD-*phosphotransferase gene, while a 297 bp deletion was observed in the reference

Detailed analysis of the CPS loci showed that 6B variants contained an intact allele of the *licD*-family phosphotransferase gene, whereas the reference contained a 300 bp gene deletion (Fig. 5). The *licD*-family phosphotransferase is involved in lipopolysaccharide biosynthesis [26]. The CPS loci of the 6B variants were phylogenetically related to subtype III lineage (6B-III) isolated from invasive disease in the Netherlands [27]. However, the variants belonged to genotypes that were distinct from 6B-III based on multilocus sequence typing (Table 2), suggesting that different clones are circulating in Malawi, and their phenotype is currently not known.

**Table 2:** Multilocus sequence typing [46] of Malawian CPS locus variants

				MLST house keeping genes							
Strain ID	Serotype	Country sampled	Sequence Type (ST)	*aroE*	*gdh*	*gki*	*recP*	*spi*	*xpt*	*ddl*	Closest Matching ST*
JF911504.1	6B-III	Netherlands	Novel	7	6	1	8	6	1	14	
JF911507.1	6B-III	Netherlands	90	5	6	1	2	6	3	4	
ERS096173	6B	Malawi	Novel	2	8	1	10	17	1	19	2863 (33D) (DLV)
											4441 (6C) (DLV)
											5266 (22A) (DLV)
ERS096172	6B	Malawi	Novel	1	43	29	1	77	1	14	8783 (NT) (DLV)
ERS096166	6B	Malawi	Novel	10	9	4	5	15	20	28	6382 (6A) (SLV)
											7744 (6A) (SLV)
ERS096165	6B	Malawi	Novel	10	9	4	5	15	4	2	6382 (6A) (DLV)
											7744 (6A) (DLV)
ERS096169	6B	Malawi	Novel	54	5	4	5	36	142	269	8050 (6A) (SLV)
ERS096157	19A	Malawi	2062	1	5	53	32	14	20	199	
ERS096159	20	Malawi	Novel	13	5	4	5	6	28	168	5392 (20) (DLV)
											7651 (20) (DLV)
ERS096158	20	Malawi	5435	2	5	36	12	6	20	269	

JF911504.1 and JF911507.1 are 6B-III subtypes previously described in Europe [27]. Serotypes with sample accession numbers ERS096165 –ERS096173 represent carriage CPS locus variants of serotype 6B in Malawian children. All the Malawian 6B variants belonged to 6B-III subtypes based on phylogeny (data not shown). Also shown are Multilocus sequence typing [46] profiles of serotypes 19A and 20 CPS locus variants. *Reference information obtained from MLST database (speumoniae.mlst.net). SLV and DLV represent single locus variant or double locus variant respectively. ST represents sequence type

### Analysis of serotype 19A and serotype 20 CPS locus variants

**Fig. 6:**
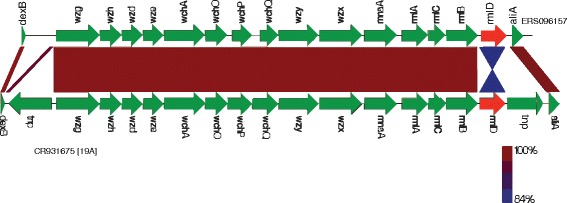
A comparison of the CPS locus sequence between serotype 19A variant and wild type. These variants were initially detected by microarray as having unusual CPS genes and were further analysed by whole-genome sequencing. The CPS locus sequences were compared between variants of serotype 19A and reference sequences CR931675. The divergent gene(s) for the variant are highlighted in red. The CPS loci of serotype 19A variant [ERS096157] showed an inversion in *rmlD* gene

**Fig. 7:**
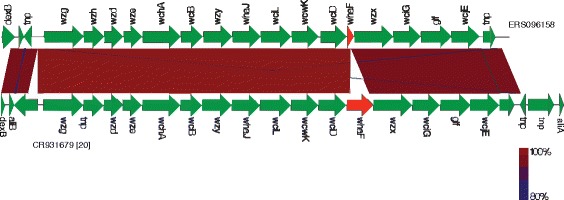
A comparison of the CPS locus sequence between serotype 20 variant and wild type. These variants were initially detected by microarray as having unusual CPS genes and were further analysed by whole-genome sequencing. A comparative analysis of the CPS locus sequences between variants of serotype 20 and reference sequences CR931679 was performed using EasyFig [47]. The divergent gene(s) for the variant are highlighted in red. The serotype 20 variant [ERS096158] contained a 717 base pair gene deletion within the *whaF* gene compared to the reference (CR931679)

The 19A variant showed an inversion in the *rmlD* gene (Fig. 6). The *rmlD* gene, together with three other genes (*rmlA, rmlB, rmlC*) encode for proteins involved in the synthesis of rhamnose sugar [28], a component of the wild type 19A polysaccharide capsule [5]. The inversion may alter the expression of the *rmlD* gene, which may affect the synthesis or change the molecular structure of the polysaccharide capsule. The pattern of SNPs within the 19A CPS locus variant and *rmlD* gene inversion was identical to serotype 19A-I subtype [27]; however, the sequence types were different (Table 2) suggesting circulation of a different clone in Malawi. The CPS locus variant of serotype 20 displayed a 716 bp deletion within the *whaF* gene (Fig. 7). This gene is located between the *wciD* and *wzx* genes and encodes for putative glycosyl transferase (GT) protein, involved in linking sugar molecules to generate a repeat unit [5].

## Discussion

This study characterised the diversity of pneumococcal carriage in Malawian children by employing a sensitive microarray serotyping method. We observed a much higher diversity of pneumococcal serotypes in Malawian children than reported elsewhere in Africa [29], where serotyping was by a less sensitive latex agglutination assay. The higher serotype diversity in Malawian children could be attributed high rates of multiple carriage and by the use of a more sensitive method of identification. We also observed that carriage of NVT represented a high proportion (~40 %) of all serotypes circulating in Malawi. These NVTs may ultimately increase in the Malawian population due to serotype replacement following the recent introduction of PCV13 [11], potentially leading to an increase in NVT IPD cases [12, 30]. A recent study in Germany has reported an increase of IPD due to NVT serotypes 15A and 23B post PCV13 [31]. Serotype 15A was detected in our dataset (Fig. 1) and it would be important to monitor the prevalence of serotype 15A and other NVT serotypes in both carriage and IPD in Malawi, post PCV13. We have shown, statistically, that younger children (0–2 years) carried a significantly higher proportion of PCV13 serotypes compared to older children (3–13 years) (*p* = 0.028). This supports observations from a recent carriage study in Kathmandu, Nepal where 44.4 % (132/297) of the pneumococcal serotype positive swabs from children aged 0–24 months contained one or more PCV13 serotypes [32]. This finding suggests that children in this age group are a reservoir of vaccine serotypes. Targeting this group for pneumococcal conjugate vaccination would therefore prevent the spread of vaccine serotypes within the community, thereby ensuring herd immunity.

Carriage of NT strains represented <1 % of all the carried population detected in Malawian children. Three categories of NTs have been reported recently [33] based on (i) complete deletion of the *cps* gene cluster (NT1), (ii) the sole presence of novel surface protein *nspA* gene (NT2) at the CPS locus or (iii) the presence of a conserved *aliB*-cluster (NT3) at the CPS locus. NTs are usually associated with carriage rather than IPD [34], which could explain why they are not included in the current vaccine formulations. However, a recent study reported that the highest rates of genetic recombination occurred in NT pneumococcal strains, which suggests their potential importance in genetic exchange events as well as species adaptation [9]. Although children in Malawi carried only a small proportion of NTs (<1 %), the ability by NTs to recombine readily may be central to the spread of antibiotic resistance, which could have a negative impact on disease control efforts.

At 40 %, we have demonstrated that the rate of multiple carriage among Malawian children is as high as has been reported elsewhere [4, 35]. Multiple carriage is thought to promote the horizontal gene transfer of antibiotic resistance and virulence genes [8, 36–38], which may contribute to the pathogen adaptation and increased risk of disease in the host. A recent report suggests that multiple carriage may promote co-infection with two or more pneumococcal serotypes [39]. It is therefore important to understand the dynamics of multiple carriage in a given setting in order to control pneumococcal spread and disease.

We did not find any statistically significant difference in the prevalence of multiple carriage between HIV negative and HIV positive children, which is similar to our recent report in adults [14]. To date, the effect of HIV infection on pneumococcal carriage is currently not fully understood; hence further studies on much larger datasets need to be conducted to address such questions. Although pneumococcal carriage rates have been shown to decrease with age [40], this study could not establish the association between age and the prevalence of multiple carriage in children and further studies are therefore recommended.

The polysaccharide capsule is essential for pneumococcal survival and transmission within the host by acting as a barrier to phagocytic killing [41]. The capsule is also a target for current conjugate vaccine formulations. One of the key mechanisms by which *S. pneumoniae* survives the host immune response and the effect of vaccination is to alter its CPS locus, through mutations and genetic recombination. We detected naturally occurring CPS locus variants in vaccine-associated serotypes 6B, 19A and 20. Although multiple carriage is reported to promote genetic recombination through horizontal gene transfer [42], it is not clear whether this played a role in altering the CPS genes of vaccine serotypes reported here.

Serotype 6B causes 10 % of IPD in young children globally and ranks second after serotype 14 [24]. In Malawi, serotype 6B is the second most isolated strain from invasive disease in children after serotype 1 [25]. In our data set, serotype 6B variants were genetically distinct from wildtype 6B serotypes. They demonstrated a significantly high SNPs density (Fig. 4a) and genetic recombination events (Fig. 4b), suggesting carriage of a different lineage of serotype 6B in Malawi. The 6B variants also had novel sequence types, and contained an insertion of the *licD*-family phosphotransferase gene (Fig. 5). To ascertain the potential behaviour of the 6B variants under vaccine pressure as the national programme expands, further work in mouse models is recommended.

The *cps* locus variant of serotype 19A showed an inversion in the *rmlD* gene (Fig. 6). Although inversions do not change the genetic composition of the sequence, recent findings suggest gene inversions may actually lower the expression level of the affected gene, resulting in abnormalities at phenotypic level [43]. In our setting, the *rmlD* gene inversion in the 19A variant may impair the functionality of the whole *rml* gene cluster necessary for rhamnose biosynthesis, a component of the polysaccharide capsule. This could lead to the production of an altered 19A capsule, which may not be recognised by the vaccine.

A structural difference of the polysaccharide capsule within serogroup 20 has previously been reported [44]. This structural difference was due to a truncation and loss of function of the *whaF* gene [44]. The truncation in the *whaF* gene correlated with the loss of an αGlc residue in the capsular polysaccharide repeat unit of serotype 20A [44]. The Malawian serotype 20 variants contained a 716 bp deletion within the *whaF* gene (Fig. 7). This deletion would render the *whaF* gene non-functional, leading to a loss of an αGlc residue in the capsular polysaccharide repeat unit of the variant. It is therefore likely that the serotype 20 variants circulating in Malawi belong to subtype 20A. However, it is unclear how this deletion affects the ability of the 20 variant to colonise and cause invasive disease, although harbouring an intact allele of the *whaF* gene has been associated with invasive strain strains of subtype 20B [44].

This study had some limitations, which made it impossible to draw some conclusions from the analysis. Because of the limited sample size, we were not able to characterise serotype-specific associations in multiple carriage. To address such limitations, a follow up study with additional samples would be recommended in this population. The microarray technique employed has limited ability to discriminate closely related serotypes, which are detected as a group, however this limitation is common to all known phenotypic and genotypic serotyping methods [45]. In addition, microarray cannot differentiate NTs from the Mitis-group Streptococci on pneumococcal positive samples [32], which may lead to an inaccurate estimation of NTs in carriage.

## Conclusions

The aim of the study was to characterise the circulating carriage profile and distribution of pneumococcal serotypes in Malawian children, by microarray.

The data clearly showed that Malawian children are exposed to a broad range of serotypes. We have shown that a large proportion of vaccine serotypes were detectable in younger children who represent the primary target group for PCV13. In particular, the high carriage rate of non-vaccine serotypes has the potential to drive serotype replacement with increasing and widespread usage of PCV13, in Malawi, based on the evidence of increasing IPD cases caused by non-vaccine serotypes 15A and 23B in Germany post PCV13 [31]. Multiple carriage is also common, and has the potential to generate further serotype (CPS) variants through horizontal gene transfer. The variants in this study also reflect naturally occurring variations. Selective pressure from vaccination may exacerbate CPS locus genetic variations and could ultimately promote vaccine escape.

To our knowledge, this is the first study to report such pneumococcal serotype diversity and rates of multiple carriage in Malawian children. The data generated provide a good scientific baseline for measuring the impact of vaccine introduction in Malawi, and also for predicting which serotypes may emerge post vaccination. Such information will be invaluable for vaccine policy.

## Abbreviations

CPS: Capsular polysaccharide
NT: Non-typeable
NVT: Non vaccine type
SNPs: Single nucleotide polymorphisms
VT: Vaccine type
MLST: Multilocus sequence typing
